# Optimal application of compressive palatal stents following mesiodens removal in pediatric patients: A Randomized Controlled Trial

**DOI:** 10.4317/medoral.24802

**Published:** 2021-10-27

**Authors:** Hyo-Jin Jang, Youn-Kyung Choi, Eun-Young Kwon, Na-Rae Choi, Yoon-Sun Jang, Jae-Min Song, Sang-Hun Shin

**Affiliations:** 1 (Bio)medical Research Institute, Pusan National University Hospital, Busan, Republic of Korea; 2 Department of Orthodontics, School of Dentistry, Pusan National University, Yangsan, Republic of Korea; 3 Department of Periodontology, Dental Clinic Center, Pusan National University Hospital, Busan, Republic of Korea; 4 Department of Oral and Maxillofacial Surgery, Pusan National University Hospital, Busan, Republic of Korea; 5 Department of Oral and Maxillofacial Surgery, School of Dentistry, Pusan National University, Yangsan, Republic of Korea; 6 Dental and Life Science Institute, School of Dentistry, Pusan National University, Yangsan, Republic of Korea; 7 Dental Research Institute, Pusan National University Dental Hospital, Yangsan, Republic of Korea

## Abstract

**Background:**

There is no scientific evidence supporting the choice of a palatal stent in patients who underwent removal of an impacted supernumerary tooth. We aimed to investigate the effects of palatal stents in patients who underwent supernumerary tooth removal through a palatal approach and to suggest the optimal stent thickness and material.

**Material and Methods:**

We recruited 144 patients who underwent extraction of a supernumerary tooth between the maxillary anterior teeth. Subjects were assigned to a control group (CG) or one of four compressive palatal stent groups (CPSGs) classified by the thickness and material of the thermoplastic acrylic stent used. Palatal gingival swelling and objective indices (healing, oral hygiene, gingival, and plaque) were evaluated before surgery and on postoperative days (PODs) 3, 7, and 14; pain/discomfort and the Child Oral Health Impact Profile (COHIP) were assessed as subjective indices of the effects of the stent.

**Results:**

The CPSGs showed faster healing than did the CG on PODs 7 (*P*<0.001) and 14 (*P*=0.043); swelling was measured by 1.64±0.88 mm and 4.52±0.39 mm, respectively. Although swelling was least in the 4-mm hard group (0.92±0.33 mm), the difference compared with that in the 2-mm hard group (1.01±0.18 mm) was not significant (*P*=0.077). The CPSGs showed better COHIP (*P*<0.001-0.036) and pain scores (*P*<0.001) than did the CG on PODs 1-3.

**Conclusions:**

Compressive palatal stents reduce discomfort by decreasing pain and alleviating swelling. Although a stent is effective regardless of its thickness and material, 2-mm hard stents maximized such positive effects with minimal discomfort.

** Key words:**Supernumerary tooth, tooth Extraction, postoperative care, oral Health, pediatric dentistry.

## Introduction

Techniques involving the incision and dissection of the upper palatal mucosa and bone reduction are common in the field of oral maxillofacial surgery for the treatment of a variety of diseases, including tumors and facial deformities (1). The most common condition requiring such techniques is a mesiodens, which accounts for 60% of all cases of supernumerary teeth (2). Indeed, although its prevalence ranges from 0.15% to 1.9%, this percentage is gradually rising (3). Various complications are associated with a mesiodens, including diastema between the central incisors, abnormal tooth eruption, abnormal occlusion, delayed eruption, displacement, rotation and root absorption of adjacent teeth, and cystic lesions (4,5). Given that children have been undergoing dental examinations at an earlier age, supernumerary teeth are being removed at an increasingly younger age (6).

Considering that removal of a mesiodens damages soft tissues and usually bones as well, palatal tissues should be stabilized by appropriately attaching the soft tissues to the bones to facilitate healing (7). In contrast to adults, both the removal of supernumerary teeth and postoperative care are more challenging in children, as they tend to be less cooperative. Similarly, younger children have more difficulty in enduring the pain and discomfort caused by the surgery (8). Therefore, application of a palatal stent following the removal of a mesiodens can protect the surgical site, facilitate hemostasis, and alleviate the swelling.

Although previous studies on supernumerary teeth focused on their causes, diagnostic analyses, and complications (2-6), intraoral stents have generally been used for treatment (e.g., following fibrosis and fistula surgeries (7,9-12), as a splint for temporomandibular joint dysfunction (13,14), or to cover a defective area in the lips and palate (15,16)), prevention (e.g., during radiotherapy (17) or as a mouth guard to prevent trauma (18)) or aesthetics (e.g., orthodontic palatal expansion stent (19) or as a guide for placing a dental implant (20)). Several studies including numerous cases have verified the material used (i.e., acrylic blocks) as well as the usefulness and safety of stents for such purposes (9,12,13,18-20), but studies on their application following the removal of supernumerary teeth are rare. Thus, the optimal material and thickness for such use have not yet been identified.

In this study, we hypothesized that the use of a palatal stent is effective in enabling rapid healing by protecting the surgical site from irritation and reducing pain and discomfort in the patient by compressing the edema.

Therefore, in this study, we aimed to investigate the effects of using a palatal stent in patients who underwent removal of an impacted supernumerary tooth through a palatal approach. Specifically, the effects of a surgical stent applied after both the incision and dissection of the palatal mucosa as well as palatal bone reduction were determined. Our primary objectives is to assess the effectiveness of the palatal stent after the removal of palatal mesiodens and secondary objectives is to evaluate the optimal thickness and the materials of the stent.

Materials and Methods

- Patients

This study is a double- blind, randomized clinical trial and aimed to confirm the effectiveness of the use of palatal stents. A total of 144 patients who underwent extraction of a supernumerary tooth between the upper incisors at the Department of Oral Maxillofacial Surgery at the Pusan National University Hospital between June 2018 and September 2019 were enrolled. The sample size for our study was computed using the G*Power 3.1 program, and the minimum sample size was calculated to be 130 (effect size 0.25, significance level 0.05, power 0.08). Written informed consent was obtained from the participants before the start of the investigation, and this study was approved by the Hospitals Ethics Review Committee (H-1906-003-079) and performed in accordance with the Declaration of Helsinki.

The inclusion criteria were the following: 1) age <12 years, 2) presence of one supernumerary tooth between the upper incisors, and 3) absence of cystic lesions other than those involving the supernumerary tooth. Thereafter, participants were assigned to the control group (CG; n=32) or one of the four compressive palatal stent groups (CPSGs) as follows: 2-mm hard acrylic group (n=30), 2-mm soft acrylic group (n= 28), 4-mm hard acrylic group (n=27), and 4-mm soft acrylic group (n=27).

**Figure 1:**
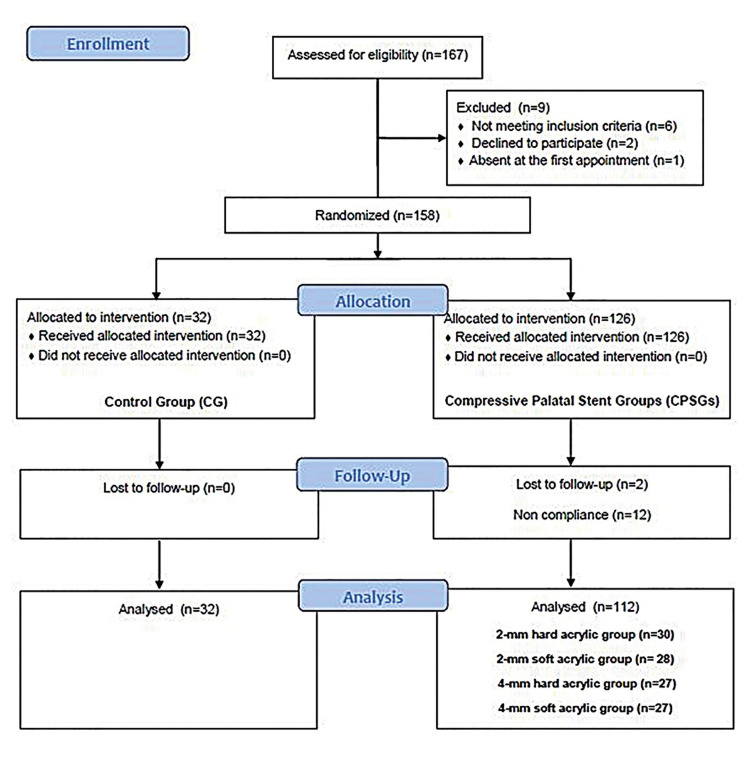
Flow chart.

Once the participant agreed to the study protocol, written informed consent was obtained. Randomization function (RAND) in Microsoft Excel (Microsoft, Redmond, WA, USA) was used to automatically assign random numbers to the qualified patients. Patient’s names were written with numbers and set in sealed envelopes, which were kept by the central trial coordinator (Jang HJ.), who was responsible for assigning the envelope numbers upon operator’s request. The subjects’ participation protocol is shown in the Consolidated Standards of Reporting Trials flow chart (Fig. 1).

Blinding of participants and the operator was feasible due to the nature of the intervention. The outcome examiner was blinded to the subject’s assigned intervention. Patient’s informations were concealed by the central trial coordinator prior to the measurement assessments. The central trial coordinator(Jang HJ) was the only one responsible for the management of all documents. Other investigators did not have the access to the documents while the coordinator was excluded from any clinical assessment. When patients came for the follow-up, the coordinator removed the stent then the patients were sent to each department for the treatment. Measuring the variable was done under the condition of which patients and the investigator did not know the groups they belonged to.

The surgery was performed by same protocol including the flap incision and elevation, osteotomy around the crown, odontomy, luxation and root extraction under general anesthesia by one surgeon of Department of Oral and Maxillofacial Surgery (Song JM). After the tooth was extracted, the flap was sutured passively, without tension, with 4/0 coated VICRYL sutures (VCP316, ETHICON). All patients were received verbal and written post-operative instructions including medication, soft diets and complications. Patients were prescribed oral analgesics (acetaminophen 10mg/kg) with antibiotics (amoxicillin hydrate/potassium clavulanate diluted 20-40 mg/kg) for 3 days.

- Fabrication of compressive palatal stent

Before surgery, an intraoral maxillary impression was taken using alginate impression materials. Subsequently, a compressive palatal stent was fabricated using thermoplastic acrylic with two different thicknesses (i.e., 2 and 4 mm) and two types of material (i.e., hard and soft acrylic material). The CPSGs wore the stent immediately after surgery until postoperative day (POD) 7, except for meal times.

- Variable measurements

Two specialists visually inspected the oral site and used the tool developed (21,22) to quantify the healing index(measured 7 and 14 days after surgery). The intraclass correlation coefficient for interrater reliability was measured and reported to be 0.92.

**Figure 2:**
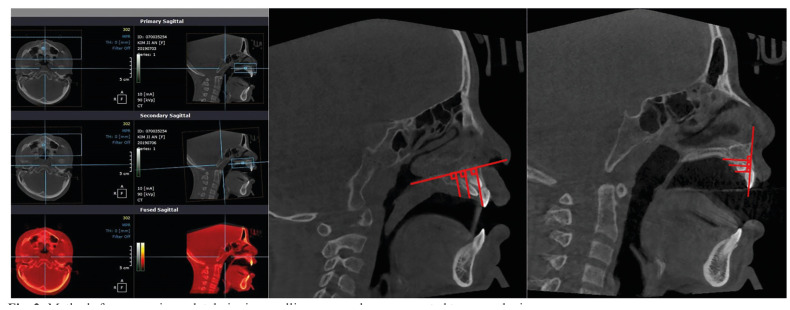
Methods for comparing palatal gingiva swelling on cone beam computed tomography images.

For measurement of palatal gingival swelling, one dentist measured the thickness of the palatal gingiva using cone beam computer tomography (CBCT) images taken before and 3 days after surgery and analyzed using image analysis software (OnDemand 3D; Cybermed Inc., Seoul, Korea). Preoperative and postoperative images of the maxilla were superimposed based on palatal plane, from the anterior nasal spine (ANS) to posterior nasal spine (PNS), using the software's auto-registration function. The vertical line (i.e., the distance to the soft tissue by making the right end, left end, and center of the mesiodens fall at 90° to the reference line drawn from the posterior tip of the palatal bone to the ANS) was measured, and the average of three measurements was used. The horizontal line (i.e., the distance to the soft tissue by making the top, middle, and bottom of the mesiodens fall at 90° to the reference line drawn from the ANS to the tip of the incisor) was measured, and the average of three measurements was used (Fig. 2).

Based on the symptoms observed in the children, caregivers were instructed to complete the Child Oral Health Impact Profile (COHIP) (23) and Questionnaire of Oral Discomfort (24) every day from PODs 1 to 7. Specifically, a five-point Likert scale was used (never [0], occasionally [1], neutral [2], generally [3], and always [4]). Pain was assessed using a visual analog scale (VAS) (measured 1 to 7 days after surgery), and the oral hygiene, gingival, and plaque indices were calculated (measured before surgery and 3, 7, 14 days after surgery).

A double-blind design was used to collect data; both participants and investigators were blinded to the experiment.

- Statistical Analysis

The collected data were analyzed using the SPSS version 22.0 software (IBM Corp., Chicago, IL, USA). The Kolmogorov-Smirnov test was performed, and homogeneity was tested using both the Chi-square test and the independent t-test. The dependent variables were analyzed using analysis of variance and the independent t-test. Bonferroni post hoc tests were conducted. The interrater reliability was analyzed using the intraclass correlation coefficient. A *P* value of 0.05 or smaller was considered significant.

## Results

The age range of the participants in this study was 5-9 years(CPSG = 7.19±2.48, CG = 7.34±2.65). More boys than girls were recruited in the CPSGs and CG (male to female ratio =1.95:1; *P*=0.774).

- Accelerated healing

A significant differences were found with quicker healing in the CPSGs than in the CG (3.04±0.56 and 2.1±0.31, respectively, on PODs 7 [P<0.05] and 14 [P<0.05]) with the healing stage over time. The healing stage according to the thickness and material of the acrylic stent, statistically significant differences were observed among all five groups (*P*<0.05). Specifically, the quickest healing was observed in the 2-mm hard group, followed by the 4-mm hard group, the 4-mm soft group, the 2-mm soft group, and the CG .

- Decreased swelling

Among the CPSGs, the 4-mm hard group had the least amount of swelling (0.92±0.33 mm), followed by the 2-mm hard group (1.01±0.18 mm), the 4-mm soft group (2.10±0.45 mm), and the 2-mm soft group (2.79±0.50 mm) (*P*<0.05) (Table 1).

**Table 1:**
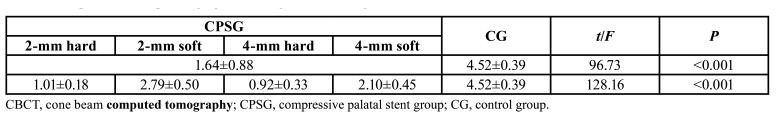
Comparison of the palatal gingiva swelling on CBCT images.

- Lessening of pain and oral discomfort

In CPSG, there was significantly less pain compared to CG from 1 to 3 days of PODs (*P*<0.05), and there was no difference after 3 days. (*P*>0.05) According to the thickness and material of the acrylic stent, although pain decreased over time in all groups, the 4-mm hard group showed statistically significantly higher pain compared to other groups. (*P*<0.05)

**Figure 3:**
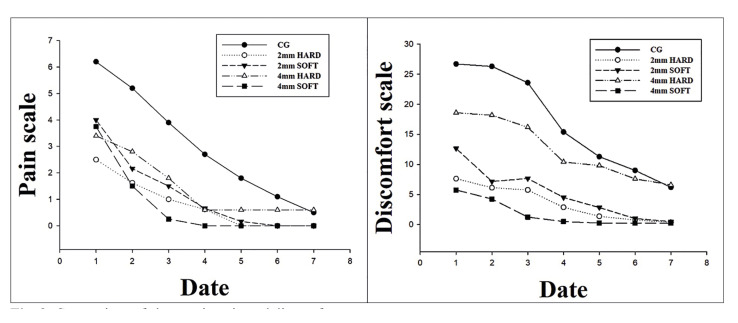
Comparison of changes in pain and discomfort.

Discomfort was observed from lowest to highest: 2-mm soft <4-mm soft <2-mm hard <4-mm hard <CG. The 4 mm hard group and CG showed similar levels of discomfort over time (*P*>0.05) (Fig. 3).

- Increase in oral health-related quality of life

**Figure 4:**
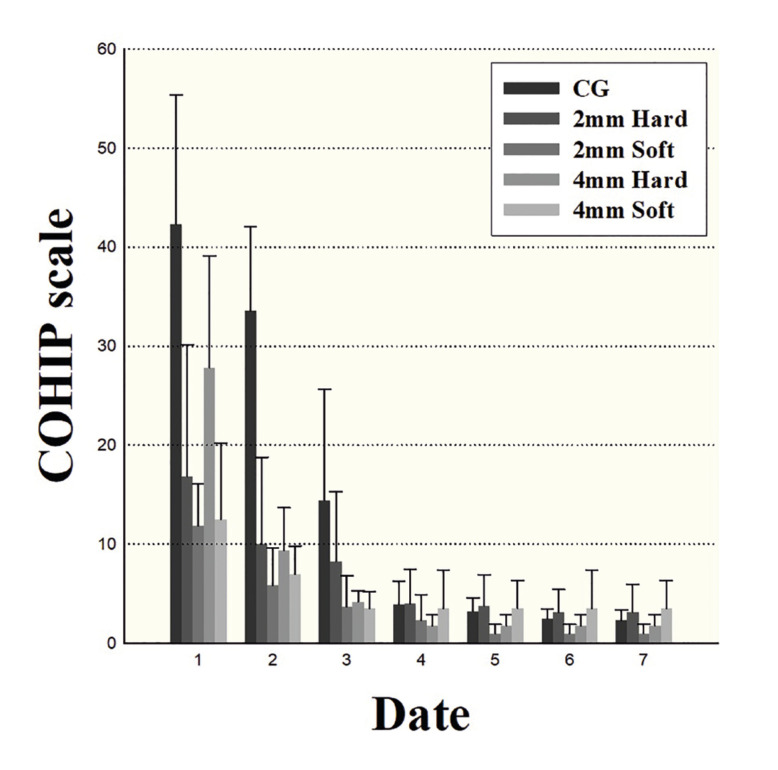
Changes in Child Oral Health Impact Profile (COHIP) over time.

On PODs 1 to 3, the CPSGs presented a better quality of life (QOL) than did the CG (*P*<0.05); however, a significant difference was not observed after POD 4 (*P*>0.05). Regarding quality of life (QOL) according to the thickness and material of the acrylic stent, no statistically significant differences were observed among the groups (*P*=0.132-0.999), even though the QOL was observed to decrease in the following order: 2 mm soft > 4 mm soft > 2 mm hard > 4 mm hard (Fig. 4).

- Improvement in the oral hygiene, plaque, and gingival indices. The descriptive statistics are shown in Table 2.

**Table 2:**
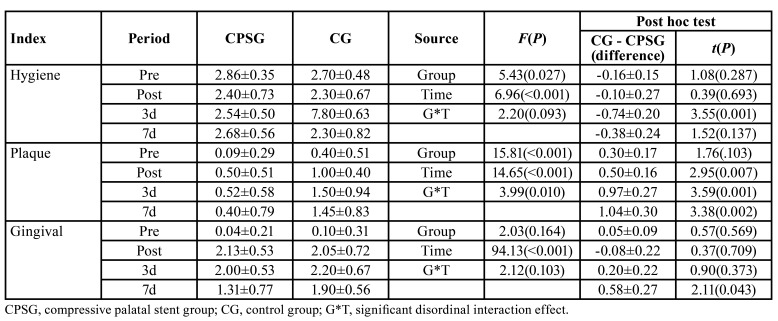
Comparison of hygiene, plaque, and gingival indices.

## Discussion

This study aimed to investigate the effects of CPS in patients (age range 5-9 years) who underwent removal of an impacted supernumerary tooth through a palatal approach. More than 60% of patients reported in previous studies underwent mesiodens extraction before the age of 14 years. Indeed, children in this age group tend to be sensitive and have difficulty in enduring the pain and discomfort caused by surgery. Therefore, clearly identifying the effects of CPS and proposing guidelines for this age group are essential to both ensure the use of appropriate appliances and expand their scope.

While acrylic (9,12,13,18-20), resin (11,14,15,17), aquasplint (7), titanium (10), and silicone (16) have been used previously for stents, thermoplastic acrylic was employed in the current study for the following reasons: 1) it can adhere to the underlying periosteum and bone tissue; 2) it can protect the surgical area ; 3) it can prevent hematoma and dead space formation; 4) it is robust enough to withstand activities of daily living; 5) it is easy to fabricate and repair, which makes it cost-efficient; and 6) it is easy to use, thereby increasing its suitability as a surgical device (9,12).

In accord with previous studies, we found that the CPSGs showed quicker healing of palatal tissues compared with the CG, which is attribuTable to the fact that the stent physically blocks contaminants (e.g., saliva and food), controls bleeding by protecting the wound from trauma, and protects the clot (25,26). In addition, we suggest that the stent prevents differentiation of granulation tissue without hindering cell recruitment (26). However, our findings differed from those of previous studies as we found that covering the periodontium interferes with the healing of the wound. This may be because we used an acrylic product with different physical and chemical properties and that the stent was worn for only 7 days. Therefore, the CPS can be considered to contribute to early mucosal healing.

In contrast to the CG, less swelling was reported in the CPSGs, suggesting that the CPS sufficiently compresses the soft tissues, which are thus attached firmly to the palate. In addition, although the CPS reduced swelling regardless of its thickness and material, stronger adhesions and less swelling were observed for the hard material than for the soft stents with the same thicknesses. However, patients were most comforTable with the 2-mm hard stents, possibly owing to the fact that a 4-mm disocclusion causes a considerable foreign body sensation and may worsen discomfort while performing basic functions such as swallowing and breathing.

Pain and oral discomfort were maintained at similar levels and patterns from POD 4 in the 4-mm hard group and the CG, whereas the remaining groups had almost no pain and discomfort. This may be explained by the pattern associated with postoperative acute pain, such that mucosal and periodontal ligament injuries and ischemia caused by atrophy induce somatic pain that subsides 72 hours later (27). Therefore, the CPS (which functions as a splint) alleviates pain by providing support; however, it causes discomfort once the pain is relieved. This indicates that the CPS had excellent preventive effects against contamination and trauma to the wound as well as analgesic effects in the early phase of healing.

With respect to the oral health-related QOL, the CPSGs had better scores than the CG before POD 3, but no difference after POD 4. This is because pain increases up to 72 hours after surgery, so it is useful to wear a CPS until POD 3 to protect the surgical site from irritation to reduce oral pain and discomfort. However, as indicated by the lowest QOL in the 4-mm hard group, wearing a CPS works just as well, suggesting that there is no need for thick and hard materials. The CPSGs also showed better oral hygiene and plaque indices than CGs at POD 3. This is inferred as a result of CPSGs enabling daily activities earlier than CGs, such as tooth brushing, with less swelling and reduced pain and discomfort, as seen in the above-mentioned results.

Overall, wearing the CPS used in the present study following surgery improves safety by reducing both swelling and pain. However, this effect lasts only until POD 3. Given that the 2-mm materials are sufficiently effective, thicker materials that cause discomfort are not recommended, although soft materials are also not suggested in cases requiring compression through close contact.

There are several limitations in the interpretation of the study. First, there is a difference in the amount of bone preparation according to the intraosseous location of the supernumerary tooth and the surrounding anatomical structures (adjacent teeth, incisive canal, etc.). The amount of osteotomy of the lesion can affect the degree of post-operative bleeding, swelling and pain. Therefore, further study is needed with the consideration of the location of impacted tooth. Secondly, our study is only limited to the patients with supernumerary tooth. However, the sample may include other diseases, such as cysts and benign tumor of the palate, to reach the generalization of the effectiveness of the stent use.

Conclusion

Palatal stents placed following both an incision of the oral mucosa and palatal bone reduction are helpful for lowering pain and discomfort by reducing swelling. In addition, although stents are effective regardless of their thickness and material, we recommend using the 2-mm hard material, as it maximizes the effects with minimal discomfort.
